# Safety and efficacy of different rotational speed during rotational atherectomy in coronary heart disease patients (RACE): study protocol for a randomized controlled trial

**DOI:** 10.1186/s13063-025-08834-6

**Published:** 2025-04-08

**Authors:** Jie Xu, Yu-wei Wang, Li-Kun Ma, Hao Hu, Hong-Wu Chen, Jing-Sheng Hua, Xiang-Yong Kong, Dan Li, Long-Wei Li, Jia-Wei Wu

**Affiliations:** Department of Cardiology, Division of Life Science and Medicine, The First Affiliated Hospital of USTC, University of Science and Technology of China, Hefei, 230001 China

**Keywords:** Rotational atherectomy, High speed, Low speed, Vascular calcification

## Abstract

**Introduction:**

The increasing incidence of coronary heart disease, driven by socio-economic development and population aging, poses significant challenges. Coronary calcification, a major factor complicating percutaneous coronary interventions (PCI), often necessitates rotational atherectomy (RA) for lesion preparation. However, the impact of different RA rotational speeds on procedural and clinical outcomes remains unclear. While low-speed RA (LSRA) has been suggested to reduce intraoperative slow flow, evidence is inconsistent, and the benefits of combining LSRA with high-speed RA (HSRA) are not well established. This study aims to evaluate the effectiveness of different rotational speed protocols to guide clinical practice.

**Methods and analysis:**

This single-center, randomized controlled trial will target patients with severe coronary artery calcification scheduled for RA. An estimated 210 patients will be enrolled based on sample size calculation, randomly assigned in a 1:1:1 ratio to different rotational speed protocols using a random number table. These will include a continuous low-speed rotation (LSRA) group (140,000 rpm), a continuous high-speed rotation (HSRA) group (180,000 rpm), and a high-speed to low-speed rotation (HSRA + LSRA) group (initially 180,000 rpm, followed by 100,000 rpm). The primary endpoint is the incidence of complications during RA, including coronary artery spasm, slow/no reflow, dissection, burr entrapment, guidewire fracture, and perforation. Secondary outcomes encompass intravascular imaging (IVUS or OCT) assessments (detecting calcific ring disruption and measuring the target lesion’s minimum lumen area (MLA) and minimum lumen diameter (MLD)); in-hospital cardiac death, acute stent thrombosis, and heart failure occurrences; and the 1-year incidence of major adverse cardiovascular and cerebrovascular events (MACCE).

**Discussion:**

The RACE study evaluates the impact of different rotational speeds in coronary rotational atherectomy, aiming to provide guidance for clinical practice. The findings may help standardize RA procedures and inform future clinical guidelines, improving procedural consistency and patient outcomes.

**Registration number:**

ChiCTR2300076194. Registered on September 27, 2023.

## Introduction

With the progression of socio-economic development and an aging population, the incidence of coronary heart disease (CHD) is steadily increasing. Against this backdrop, the issue of coronary artery calcification (CAC) has become increasingly prominent, especially among middle-aged and older adults. Epidemiological data indicate that in adults aged 45 to 84, the prevalence of CAC can be as high as 70.4% [1]. Among patients undergoing percutaneous coronary intervention (PCI), approximately 18 to 24% exhibit moderate to severe calcification [2]. Studies have shown that patients with severe CAC face a higher risk of major adverse cardiovascular and cerebrovascular events (MACCE), including death, myocardial infarction, target vessel revascularization, and in-stent thrombosis [3–7]. Therefore, effectively managing CAC is crucial for improving the outcomes of PCI.

In recent years, with the increasing emphasis on “optimizing PCI,” pre-procedural vessel preparation for PCI has garnered widespread attention. In this context, calcific lesions have become as a significant challenge in cardiovascular intervention. Despite advancements in PCI techniques, the presence of CAC remains a major barrier to both short-term and long-term success of treatments. Rotational atherectomy (RA) is a key technique for managing calcified coronary lesions, offering distinct clinical benefits. By modifying calcific plaques, facilitating stent delivery, and increasing the luminal area, RA enhances PCI success rates [8]. The PROTECT II trial studied RA in high-risk PCI and found that while essential for severe calcific lesions, it may also increase the risk of perioperative myocardial infarction [9]. Among procedural variables, burr rotation speed is considered a key determinant of both procedural outcomes and complication rates [10]. However, the optimal strategy for rotation speed remains undefined. Expert opinions on this issue vary across different regions and clinical practices [11–14]. Low-speed rotational atherectomy (LSRA) has been reported to reduce intraoperative slow flow, but the supporting evidence is inconsistent, and no consensus has been reached on its clinical benefits [15–17]. Recent studies have reported that a combination strategy of LSRA and high-speed RA (HSRA) is a safe and feasible approach for managing severely calcified lesions in clinical practice, yet its advantage over conventional methods remains unclear [18]. While LSRA following HSRA has been associated with greater lumen gain compared to continuous HSRA, conflicting findings exist, and its long-term impact is not well established [18, 19]. Most existing studies are retrospective or observational, and large-scale trials on the optimal rotational speed for better procedural success and outcomes are lacking.

Our previous retrospective studies have identified differences in perioperative outcomes among patients undergoing RA at different rotational speeds [10, 20]. However, the optimal and safest burr rotation speed remains uncertain, highlighting the need for further prospective research to establish a standardized approach.

To address this gap, the RACE trial aims to evaluate the safety and efficacy of different rotational speed protocols by analyzing their impact on perioperative complications and long-term prognosis in patients with CHD.

## Methods

### Study design

This is a single-center, randomized controlled study focusing on patients with CHD who have significant CAC and are candidates for RA. The objective of the study is to examine differences in perioperative complications and long-term prognosis among various rotational speed protocols in these patients. We plan to continuously recruit participants at a tertiary A-grade hospital in Anhui, China, which is renowned for its large patient population with cardiovascular diseases and advanced medical technology. Patients who require RA treatment and meet the specific inclusion criteria (Table 1) will be enrolled in the study. Prior to undergoing RA, the principal investigator (PI) will verify patient eligibility and obtain written informed consent.

**Table 1:** Eligible criteria

Inclusion criteria • Age ≥ 18 years old • The presence of severe coronary calcification was evaluated utilizing one of the following methods: ➢ CAC assessed as severe based on semi-quantitative scoring ➢ IVUS detection of calcific lesions exceeding 270° in arc ➢ IVUS identification of prominent calcific nodules protruding into the lumen ➢ OCT assessment scoring calcification as 4 points ➢ OCT identification of prominent calcific nodules protruding into the lumen ➢ Inability of the balloon to pass or fully expand the lesion
Exclusion criteria • Age > 80 years • Severe left ventricular dysfunction [LVEF ≤ 30%] • Lesions where the rotational atherectomy guidewire cannot pass • Lesions rich in thrombus • Venous bridge vascular lesions • Lesions with acute angles ≤ 90° • Severe spiral dissection • Immediate rotational atherectomy following stent placement • Pregnant or breastfeeding women • Anticipated lifespan < 1 year • Inability to comply with follow-up

*CAC* Coronary Artery Calcification, *OCT *Optical Coherence Tomography, *IVUS *Intravascular Ultrasound, *LVEF *Left Ventricular Ejection Fraction

Lesions with acute angulation (≤ 90°) were excluded as studies have identified severe angulation as an independent predictor of procedural and clinical failure in RA [21]. Immediate RA following stent placement was excluded due to the high risk of burr entrapment and suboptimal long-term outcomes reported in previous studies [22–25]. *CAC*, coronary artery calcification; *OCT*, optical coherence tomography; *IVUS*, intravascular ultrasound; *LVEF*, left ventricular ejection fraction.

It is anticipated that 210 patients who meet the inclusion and exclusion criteria will be randomly assigned in a 1:1:1 ratio to three different rotational speed groups: a continuous low-speed rotation (LSRA) group (140,000 rpm), a continuous high-speed rotation (HSRA) group (180,000 rpm), and a high-speed to low-speed rotation (HSRA + LSRA) group (initially 180,000 rpm, followed by 100,000 rpm). The study includes a 12-month follow-up period. Research protocols and documents have been distributed to relevant researchers. Recruitment began on October 1, 2023, and will continue until the estimated number of patients is reached, with the study expected to be completed within 3 years.

This study protocol has been approved by the Medical Research Ethics Committee of the First Affiliated Hospital of the University of Science and Technology of China (Anhui Provincial Hospital) under approval number 2023-KY- 076.

### Randomization

**Fig. 1:**
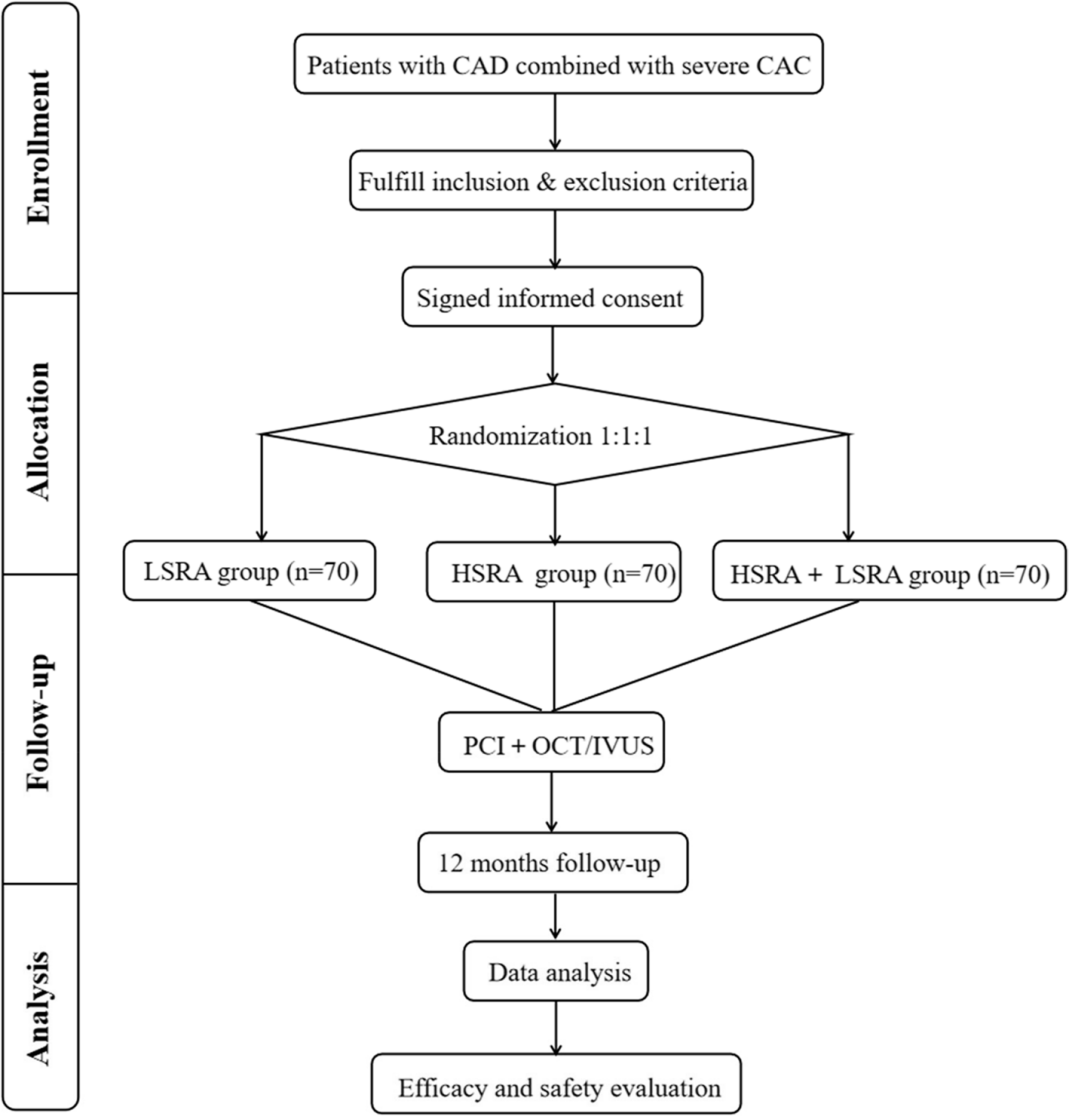
LSRA, low-speed rotation; HSRA, high-speed rotation; HSRA + LSRA, a high-speed to low-speed rotation; OCT, optical coherence tomography; IVUS, intravascular ultrasound; PCI, percutaneous

Eligible patients who provide informed consent will be randomly assigned in a 1:1:1 ratio to one of three rotational speed groups for RA treatment (Fig. 1). To ensure unbiased random allocation, a computer-generated random number table was created prior to the commencement of the study. Patients will be assigned sequentially based on this table to one of three groups: LSRA, HSRA, or HSRA + LSRA, maintaining a 1:1:1 distribution to ensure group balance. Once a patient meets the inclusion criteria, the assigned study personnel will disclose the randomization result but will not be involved in data collection or follow-up. To minimize bias, outcome assessors will remain blinded to the treatment allocation. These assessors, responsible for evaluating primary and secondary endpoints, will not be aware of the specific intervention each patient receives. Randomization data will be securely stored and managed by an independent data management team, ensuring blinding until the completion of data analysis. This methodology ensures an objective assessment of the safety and efficacy of different RA rotational speed protocols, offering valuable insights for optimizing treatment strategies in coronary heart disease.

### Clinical procedures

Our center utilizes the Siemens DTC (or PHILIPS FD1010) angiography system for coronary angiography and interventional therapy. The Rotablator System (Boston Scientific Corporation, Natick, MA, USA) was used for all the RA procedures. Prior to the procedure, patients were treated with aspirin (100 mg per day) and thienopyridine (clopidogrel 75 mg daily or ticagrelor 100 mg twice daily). In addition, normal heparin (70–100 units/kg) was administered intravenously during the procedure to achieve an appropriate activated clotting time (250 s). Percutaneous coronary intervention was performed at the operator’s discretion. In previous studies [26], we observed that higher experience in RA might be linked to worse outcome in PCI via femoral approach in both stable angina and acute coronary syndrome settings. However, the specific approach should still be determined based on the characteristics of the patient’s specific lesions and the conditions of the peripheral vascular pathways. Catheters up to 7 F in diameter were used to exchange a 0.009-inch (1 inch = 2.54 cm) rotating wire flexible disk to the distal end of the target lesion with the help of a Finecross or Crossair microcatheter. Each RA session lasted less than 30 s, with a 60-s interval between each RA. During the RA procedure, a continuous pressurized drip of plain heparin nitroglycerin flushing solution was administered. Noncompliant balloon dilation and stent placement were performed following the RA procedure, depending on the lesion characteristics. Patients who developed bradyarrhythmias after RA received both a strong cough and intravenous atropine. Atropine and a temporary pacemaker were prepared for patients with right coronary artery or systolic branch predominance. Implantation of intra-aortic balloon counter pulsation (IABP) depends on the judgment and guidance of the supervising cardiologist. Following the completion of rotational atherectomy, a follow-up coronary angiography was performed. In cases of vascular spasm, intracoronary nitroglycerin or sodium nitroprusside was administered via the catheter to relieve the spasm.

This study will assess the effectiveness of coronary RA using IVUS and OCT, which provide detailed imaging of lesion structure and treatment outcomes. IVUS is a technique that uses high-frequency sound waves to visualize the inner walls of blood vessels [27]. IVUS utilizes high-frequency sound waves to visualize the inner walls of blood vessels, allowing us to accurately measure the degree of stenosis and the composition of the plaque. In this study, IVUS will assess intimal lumen diameter and plaque morphology before and after RA. This will help clarify how different rotational speeds affect the vessel wall and plaques. On the other hand, OCT uses near-infrared light to capture high-resolution images of the vessel lining [28]. OCT can provide higher resolution images than IVUS, especially in assessing intima and plaque surface characteristics. In this study, OCT will be used to assess endovascular surface features after RA treatment, including plaque tears, stenting effects, and possible vessel damage. By combining IVUS and OCT, we will be able to comprehensively evaluate the effects of RA treatment at both macroscopic and microscopic levels. This information will provide important clinical data for evaluating the safety and efficacy of different rotational speeds, thus contributing to optimize the use of RA in the treatment of coronary artery disease (CAD).

### Planned outcomes

The primary focus of this study is to investigate the safety and efficacy of using different rotational speeds in coronary rotational atherectomy. The main endpoint is the incidence of complications during the atherectomy procedure. Secondary endpoints include assessments through intravascular imaging, in-hospital adverse cardiovascular events, and adverse cardiovascular events during the 1-year follow-up period (Table 2).

**Table 2:** Primary and secondary endpoints

Primary endpoints	
Complications during RA	Coronary artery spasm Slow flow/no reflow Dissection Burr entrapment Guidewire fracture Perforation
Secondary endpoints	
IVUS/OCT	① Calcific ring disruption ② MLA ③ MLD
Adverse events	Cardiac death Acute stent thrombosis Heart failure
MACCE	Cardiac death Non-fatal myocardial infarction Ischemia-driven revascularization TIA

In IVUS, calcific ring disruption is defined as a loss of continuity in the calcified plaque, presenting as fissures, defects, or fractures along the plaque margins. In OCT, it is identified by cracks, defects, or fractures within the calcified region, resulting in surface irregularities or cavitations. *RA*, rotational atherectomy; *MLA*, minimum lumen area of target lesion; *MLD*, minimum lumen diameter of target lesion; *TIA*, transient ischemic attack; *MACCE*, major adverse cardiovascular and cerebrovascular events; *IVUS*, intravascular ultrasound; *OCT*, optical coherence tomography.

### Trial schedule and follow-up

The follow-up plan includes both perioperative and long-term clinical outcomes (see Table 3). The clinical data to be collected will include preoperative baseline information, including demographic information, imaging studies (electrocardiogram, echocardiography), laboratory tests (including total platelet count, cardiac troponin I, and N-terminal pro-brain natriuretic peptide (NT-proBNP)), medication history, as well as primary and secondary endpoint data. Follow-ups for all participants are scheduled at 12 months, during which both primary and secondary outcomes, as well as safety, will be assessed.

**Table 3:**
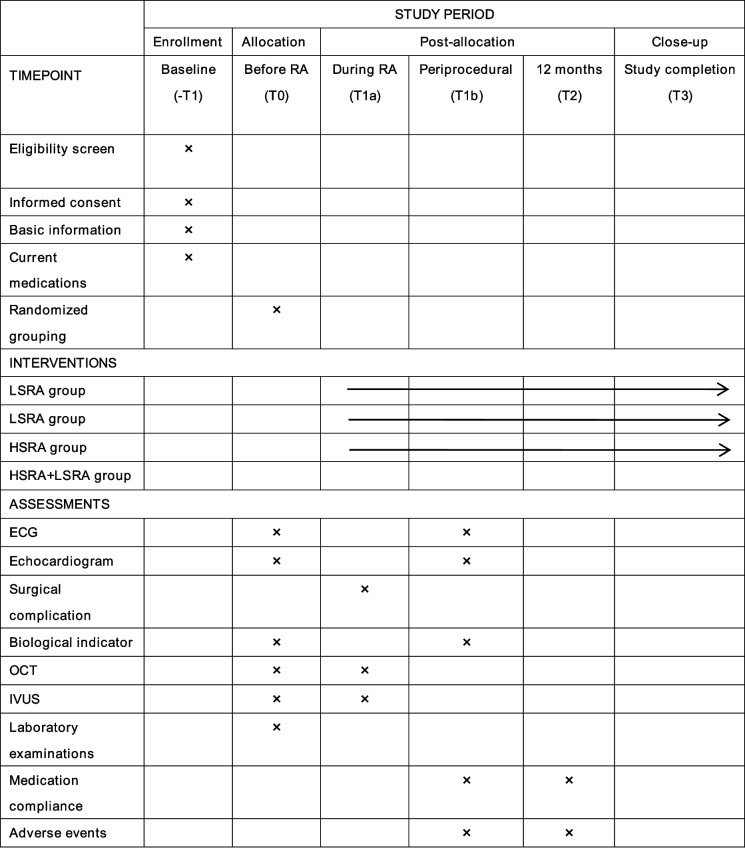
Schedule of recruitment, interventions, and assessments

*OCT* optical coherence tomography, *IVUS* Intravascular Ultrasound, *ECG* electrocardiography, *RA* Rotational Atherectomy

### Sample size

According to the literature, the probability of complications occurring during RA is 4%, 14% in the HSRA + LSRA group [18], and 24% at higher rotational speeds. A sample size of 175 achieves 80% power to detect an effect size (*W*) of 0.2353 using a 2-degree-of-freedom chi-square test with a significance level of *α* = 0.05 [29]. Considering a 15% dropout rate, the total required sample size is 210, with 70 participants per group.

### Statistical analysis

All data will be statistically analyzed using SPSS version 22.0. Quantitative data will be presented as mean ± standard deviation (± s), with intergroup comparisons made using independent sample *t*-tests. Categorical data will be presented as number (percentage) [*n* (%)], with intergroup comparisons using the χ^2^ test. Logistic regression analysis will be applied to ascertain the correlation between coronary rotational speed and perioperative complications, as well as 1-year MACCE. Considering potential data missing and loss to follow-up, we will mainly use intention-to-treat (ITT) analysis and apply per-protocol (PP) dataset for sensitivity analysis. Besides, we will preset some subgroup analyses (e.g., based on patient age groups or lesion complexity) to verify results’ stability and explore special effect participants. To account for multiple comparisons, family-wise error rate (FWER) control methods will be applied where appropriate. Specifically, Bonferroni correction (adjusting the significance threshold to *α*′ = 0.025) and the Holm-Bonferroni procedure (a stepwise approach that improves statistical power while maintaining FWER control) will be used for primary analyses. To minimize unnecessary tests, only key comparisons (HSRA vs. LSRA; HSRA + LSRA vs. LSRA) will be included. When the sample size is limited, Holm’s method will be preferred over Bonferroni to optimize the balance between statistical power and error control. A *P* value of < 0.05 will be considered statistically significant. All statistical tests will be two-sided.

### Patient and public involvement

This study is conducted without patient or public involvement in its design, execution, or analysis. Physicians will communicate the results to participants.

## Discussion

The RACE study aims to compare perioperative complications and long-term outcomes in CAD patients. These patients will undergo different rotational speed protocols during RA. The study will also assess the safety and effectiveness of these different speed protocols.

CAC is a common phenomenon in CAD, particularly prevalent among the elderly and those with chronic cardiovascular conditions. The presence of CAC complicates PCI, often reducing the effectiveness of traditional balloon angioplasty and stent placement. Approximately half of CAD patients exhibit some level of CAC, with up to 20% of those undergoing PCI presenting with moderate to severe calcification [30, 31]. Such calcification is associated with an increased rate of MACCE, target lesion revascularization (TLR), and target vessel revascularization [32–34]. RA is widely recognized as an effective percutaneous treatment for severe calcified or fibrotic lesions unmanageable by traditional PCI. As RA technology has matured, the indications for PCI have expanded, enhancing the success rate of complex coronary interventions. However, RA can result in unique complications such as slow flow, burr entrapment, and vessel perforation, which are closely linked to patient morbidity and mortality rates. Among these, slow flow/no reflow is the most frequently observed complication following RA, as reported in previous studies. The reported incidence of slow flow/no reflow ranges from 2 to 27% [17, 18, 35]. The causes of complications are not fully understood, and research on how rotational speed affects outcomes is limited. This gap necessitates further investigation to optimize RA treatment strategies and minimize complications.

Previous studies have shown that excessively high or low RA speeds can increase the risk of postoperative complications. For instance, high speeds might lead to overheating, endothelial damage, or platelet activation [16], while low speeds may be insufficient for effective calcific plaque removal. A randomized controlled trial [17] found no significant reduction in slow flow rates following low-speed RA compared to high-speed RA. A single-center, observational, retrospective study on patients with acute coronary syndrome (ACS) [10] divided patients into a low-speed group (130,000 to 150,000 rpm, 182 cases) and a high-speed group (160,000 to 220,000 rpm, 101 cases). The results showed a higher risk of vascular spasm in the low-speed ACS group and an increased risk of slow flow in the high-speed group. However, this study’s small sample size and retrospective design limit its ability to fully confirm the results of randomized controlled trials. It is important to note that our study includes all types of coronary heart disease, but lesions rich in thrombus were excluded. Furthermore, our study focuses on patients with severe coronary artery calcification, who may respond differently to RA compared to ACS patients, providing a new perspective on how different speeds influence complications and outcomes.

Besides postoperative complications, the impact of RA speeds on long-term patient outcomes is also a focal point of our study. Existing literature lacks studies on the long-term effects associated with different RA speeds. A retrospective study by Wu et al. [20] revealed that there was no significant difference in 6-month outcomes between different speeds, but there suggested an increased risk of major adverse cardiac events and MACCE with higher speeds. To explore this further, our study extends the follow-up period to 1 year, aiming to gather more comprehensive and reliable data.

The selection of rotational speeds in this study was based on current expert recommendations and clinical experience. Since no universally accepted optimal RA speed exists, guidelines vary in their recommendations [11, 12, 14]. For the hybrid approach, previous studies have used different protocols. Yamamoto et al. (190,000 → 110,000 rpm) and Kobayashi et al. (220,000 → 120,000 rpm) both found this strategy to be safe, though their results on lumen gain differed. Considering these differences, we selected 140,000 rpm, 180,000 rpm, and a hybrid approach to further assess how speed variations influence procedural success and clinical outcomes. This design enables a direct comparison between LSRA and HSRA while also examining the effects of speed transitions, particularly in complex calcified lesions.

Moreover, the introduction of intravascular imaging offers insights into the mechanisms of stent failure, emphasizing the importance of optimal lesion preparation before stent implantation [36]. Our study utilizes IVUS and OCT to collect data on calcific ring disruption, MLA and MLD of target lesions, serving as safety assessment indicators.

This study aims to explore how different rotational speeds affect the efficacy and clinical outcomes of RA. By systematically analyzing their impact, we hope to provide practical guidance for clinical decision-making and contribute to the standardization of RA procedures. The findings may serve as a reference for future clinical guidelines, helping to establish more consistent treatment strategies. Ultimately, standardization could improve procedural predictability and patient outcomes over the long term.

### Limitations

Our study has several limitations. First, it was conducted at a single medical center, which may limit the generalizability of the results. Studies conducted at different centers may yield varied outcomes due to regional differences, demographic characteristics, and available treatment facilities. Second, despite our efforts to maintain consistency in the rotational atherectomy (RA) procedure, some variability in operation remains. For example, interventional cardiologists may use different burr advancement techniques, such as rapid or slow pecking, which could impact treatment outcomes. Finally, the skill level and experience of the operators could significantly influence the study’s results. Variations in operator expertise and experience may lead to inconsistencies in treatment effectiveness. To address this, standardized procedural protocols, operator training, and quality control measures have been implemented to ensure greater consistency.

## Dissemination plan

We plan to disseminate the findings of this study through multiple channels. This includes publishing articles in peer-reviewed academic journals, presenting at relevant academic conferences, and engaging with the public, policymakers, and healthcare professionals. Additionally, we will share updates on the research progress via social media and other online platforms to facilitate the exchange and application of scientific knowledge. Efforts will be made to ensure broad dissemination of the research outcomes, with the goal of positively impacting society.

## Trial status

The current protocol version 2.1 was issued on September 22, 2024. The recruitment started with the first patient included on October 1, 2023. At the submission of this manuscript on September 22, 2024, 132 patients have been included and randomized. Recruitment is ongoing and should be completed by December 2025.

## Data Availability

The datasets analyzed during the current study and statistical code will be available from the corresponding author on reasonable request, as is the full protocol.

## Abbreviations

CHD: Coronary heart disease
CAC: Coronary artery calcification
HSRA: High-speed rotation
IVUS: Intravascular ultrasound
LSRA: Low-speed rotation
LVEF: Left ventricular ejection fraction
MLA: Minimum lumen area
MLD: Minimum lumen diameter
MACCE: Major adverse cardiovascular and cerebrovascular event
OCT: Optical coherence tomography
PCI: Percutaneous coronary interventions
RA: Rotational atherectomy
